# A New Prognostic Risk Score: Based on the Analysis of Autophagy-Related Genes and Renal Cell Carcinoma

**DOI:** 10.3389/fgene.2021.820154

**Published:** 2022-02-14

**Authors:** Minxin He, Mingrui Li, Yibing Guan, Ziyan Wan, Juanhua Tian, Fangshi Xu, Haibin Zhou, Mei Gao, Hang Bi, Tie Chong

**Affiliations:** ^1^ Department of Urology, The Second Afilliated Hospital, School of Medicine, Xi’an Jiaotong University, Xi’an, China; ^2^ School of Medicine, Xi’an Jiaotong University, Xi’an, China

**Keywords:** risk score, prognosis, bioinformactics analysis, renal cell carcinoma, autophagy

## Abstract

**Introduction:** Clear cell renal cell carcinoma (ccRCC) patients suffer from its high recurrence and metastasis rate, and a new prognostic risk score to predict individuals with high possibility of recurrence or metastasis is in urgent need. Autophagy has been found to have a dual influence on tumorigenesis. In this study we aim to analyze autophagy related genes (ATGs) and ccRCC patients and find a new prognostic risk score. Method: Analyzing differential expression genes (DEGs) in TCGA-KIRC dataset, and took intersection with ATGs. Through lasso, univariate, and multivariate cox regression, DEGs were chosen, and the coefficients and expression levels of them were components constructing the formula of risk score. We analyzed mRNA expression of DEGs in tumor and normal tissue in ONCOMINE database and TCGA-KIRC dataset. The Human Protein Atlas (HPA) was used to analyze protein levels of DEGs. The protein-protein interaction (PPI) network was examined in STRING and visualized in cytoscape. Functional enrichment analysis was performed in RStudio. To prove the ability and practicibility of risk score, we analyzed univariate and multivariate cox regression, Kaplan-Meier curve (K-M curve), risk factor association diagram, receiver operating characteristic curve (ROC curve) of survival and nomogram, and the performance of nomogram was evaluated by calibration curve. Then we further explored functional enrichment related to risk groups through Gene Set Enrichment Analysis (GSEA), weighted gene co-expression network analysis (WGCNA), and Metascape database. At last, we investigated immune cell infiltration of DEGs and two risk groups through TIMER database and “Cibersort” algorithm.

**Result:** We identified 7 DEGs (BIRC5, CAPS, CLDN7, CLVS1, GMIP, IFI16, and TCIRG1) as components of construction of risk score. All 7 DEGs were differently expressed in ccRCC and normal tissue according to ONCOMINE database and TCGA-KIRC dataset. Functional enrichment analysis indicated DEGs, and their most associated genes were shown to be abundant in autophagy-related pathways and played roles in tumorigenesis and progression processes. A serious analysis proved that this risk score is independent from the risk signature of ccRCC patients.

**Conclusion:** The risk score constructed by 7 DEGs had the ability of predicting prognosis of ccRCC patients and was conducive to the identification of novel prognostic molecular markers. However, further experiment is still needed to verify its ability and practicability.

## Introduction

Renal cell carcinoma (RCC) is a prevalent tumor of the urinary system as it was reported by GLOBOCAN in 2020, with an incidence of 2.2% and mortality of 1.8% annually (Sung et al., 2021). Clinically, the main treatment of RCC patients is radical or unitary partial nephrectomy; however, about 30% of postoperative patients have the potential to be found with recurrence or metastasis (Capitanio et al., 2019). RCC is insensitive to chemotherapy or radiation, although in recent years anti-angiogenesis molecular targeted therapy has become the standard of care for advanced RCC, and most patients have developed drug resistance after 5–11 months (Khattak and Larkin, 2014). Up to now, the diagnosis of recurrence or metastasis of RCC still relies on imaging, but it is always too late, and patients who were found recurrence or metastasis by imaging have a poor prognosis. Thus, it’s of great significance for early diagnosis and treatment of RCC patients to find new biomarkers.

Autophagy refers to the process by which lysosomes decompose cellular materials to provide cells with biosynthetic components and energy sources (Glick et al., 2010). This process has been found relevant to many human diseases (Mizushima and Levine, 2020) such as cardiovascular disease (Gatica et al., 2021), Parkinson’s disease (Lizama and Chu, 2021), Alzheimer’s disease (Zhang et al., 2021), and so on. In the process of tumorigenesis and development, researches found autophagy had dual roles; in the earlier stage autophagy inhibits tumors from happening, while in the later stage it facilitates the progression of tumor (Kimmelman, 2011; Rangel et al., 2021). Clear cell RCC (ccRCC) accounts for the majority of RCC and had poorer prognosis; as a result, our study aims to combine ccRCC and autophagy and investigate how autophagy affects ccRCC, then build a risk score and provide insight for prognosis and treatment of ccRCC.

## Materials and Methods

### Data Source

We obtained the clinical information, raw counts of RNA-sequencing data, overall survival (OS), and disease free survival (DFS) of 537 ccRCC patients and 74 paracancerous samples in the cancer genome atlas-kidney renal clear cell carcinoma (TCGA-KIRC) dataset from the TCGA database (Cancer Genome Atlas Research et al., 2013) (http://portal.gdc.cancer.gov) through R package “TCGAbiolinks” (Colaprico et al., 2016). Gene IDs conversion were finished with the assistance of a GTF file which were downloaded from GENCODE (http://www.gencodegenes.org/), and 18,569 protein-coding genes were annotated by gene IDs and were selected for subsequent analysis. To meet the requirement of data integrality, patients with the following criteria were excluded from subsequent analysis: (1) patients with OS less than 1 month, (2) patients with inadequate clinical information. Finally, a total of 515 ccRCC patients were selected for further analysis. A total of 531 autophagy related genes (ATGs) was gathered from the human autophagy database (HADb, http://www.autophagy.lu/index.html) and GO_AUTOPHAGY dataset from The Molecular Signatures Database (MsigDB) (http://www.gsea-msigdb.org/gsea/msigdb/index.jsp) (Wang Y. et al., 2020).

### Selecting DEGs

Variation analysis of gene expression in TCGA-KIRC dataset was accomplished by R package “Deseq2” (Love et al., 2014), genes with |log_2_ Fold Change| (|log_2_ FC|) ≥ 1, and adjusted *p* value < 0.05 were regarded as differentially expressed genes (DEGs). Take the intersection of DEGs and ATGs. R package “ezcox” (http://github.com/ShixiangWang/ezcox/issue/23) was used for univariate cox regression of the intersection, then genes with *p* < 0.05 in univariate cox regression underwent lasso regression and multivariate cox regression. Finally, genes with *p* < 0.05 were selected as DEGs that were selected to construct a new risk score formula.

### Analysis of mRNA and Protein Expression Levels of DEGs

mRNA expression levels of DEGs were analyzed based on the data from TCGA-KIRC dataset and visualized by RStudio. Meanwhile, we explored mRNA expression levels of DEGs in different datasets through the ONCOMINE database (Rhodes et al., 2004) (http://www.oncomine.org). The protein levels of DEGs were tested through The Human Protein Atlas (Uhlen et al., 2017) (HPA, http://www.proteinatlas.org).

### Protein-Protein Interaction Network and Enrichment Analysis

Protein-protein interaction (PPI) network analysis was finished in STRING (Szklarczyk et al., 2019) (http://string-db.org), selecting the top 50 closest genes with DEGs and visualized by cytoscape. Gene ontology (GO) and Kyoto Encyclopedia of Genes and Genomes (KEGG) enrichment analysis of DEGs and their closest genes was finished by R package “Clusterprofiler” (Wu et al., 2021).

### Construction of Risk Score

Performing multivariate cox regression of DEGs and collecting the expression levels of DEGs and their coefficients to construct the formula of risk score:

$$\mathrm{Risk}\;\mathrm{score}=\sum_{i=1}^{n}coef_{i}*Exp_{i}$$

Correlation analysis of risk score and other clinical signatures was performed by the method of “Spearman”. We divided patients from the TCGA-KIRC dataset into two cohorts, train cohort and validation cohort with R package “caret.” Depict the receiver operating characteristic curve (ROC curve), Kaplan-Meier curve (K-M curve), and risk factor association diagram of risk score and calculate its area under the curve (AUC) in train cohort. Furthermore, univariate and multivariate cox regression was performed to prove risk score as an independent risk factor of ccRCC patients. Likewise, we tested results above through data from validation cohort and total cohort. Nomogram was to predict the probability of 1-, 3-, and 5-years survival for ccRCC patients according to the results from multivariate cox regression, and calibration curves were drawn to evaluate the nomogram. We’ve published a glycolysis-related risk score signature before, since both of our studies were metabolism-related, and we then compared their ability of predicting prognosis of ccRCC patients by depicting ROC curve and circulating AUC. All analytical methods above were finished in Rstudio with R packages such as “timeROC,” “survival,” “survminer,” “rms,” and “ggrisk,” and *p* < 0.05 was considered as statistically significant.

To further explore pathways related with risk score we then performed in Gene Set Enrichment Analysis (GSEA) (Subramanian et al., 2005) in high and low risk groups; |Normalized Enrichment Score| (|NES|) ≥ 1.5, *p* < 0.05 and false discovery rate (FDR) < 0.25 were set as threshold. Additionally, to find out the genes that were connected with risk score and their function, weighted gene co-expression network analysis (WGCNA) (Langfelder and Horvath, 2008) was performed, and genes with top 5,000 median absolute deviation were analyzed and soft threshold was selected when scale free *R* ^2^ = 0.9. Genes in the most significant co-expression module were then analyzed in Metascape (Zhou et al., 2019) (http://metascape.org). The threshold of min overlap = 3, *p* value cutoff = 0.01, and min enrichment = 1.5 was set to select enriched pathways in the module. MCODE was selected with physical score >0.132, min network size = 3, max network size = 500, and databases as physical core.

### Immune Cell Infiltration

We explored immune infiltration of DEGs from TIMER 2.0 (Li et al., 2020) (https:// timer.cistrome.org/). As for the analysis of immune infiltration in ccRCC patients, “Cibersort” algorithm (Newman et al., 2015) was performed in RStudio. Also, the difference of immune infiltration in different groups of risk score was analyzed.

## Results

### Acquisition of DEGs

**FIGURE 1:**
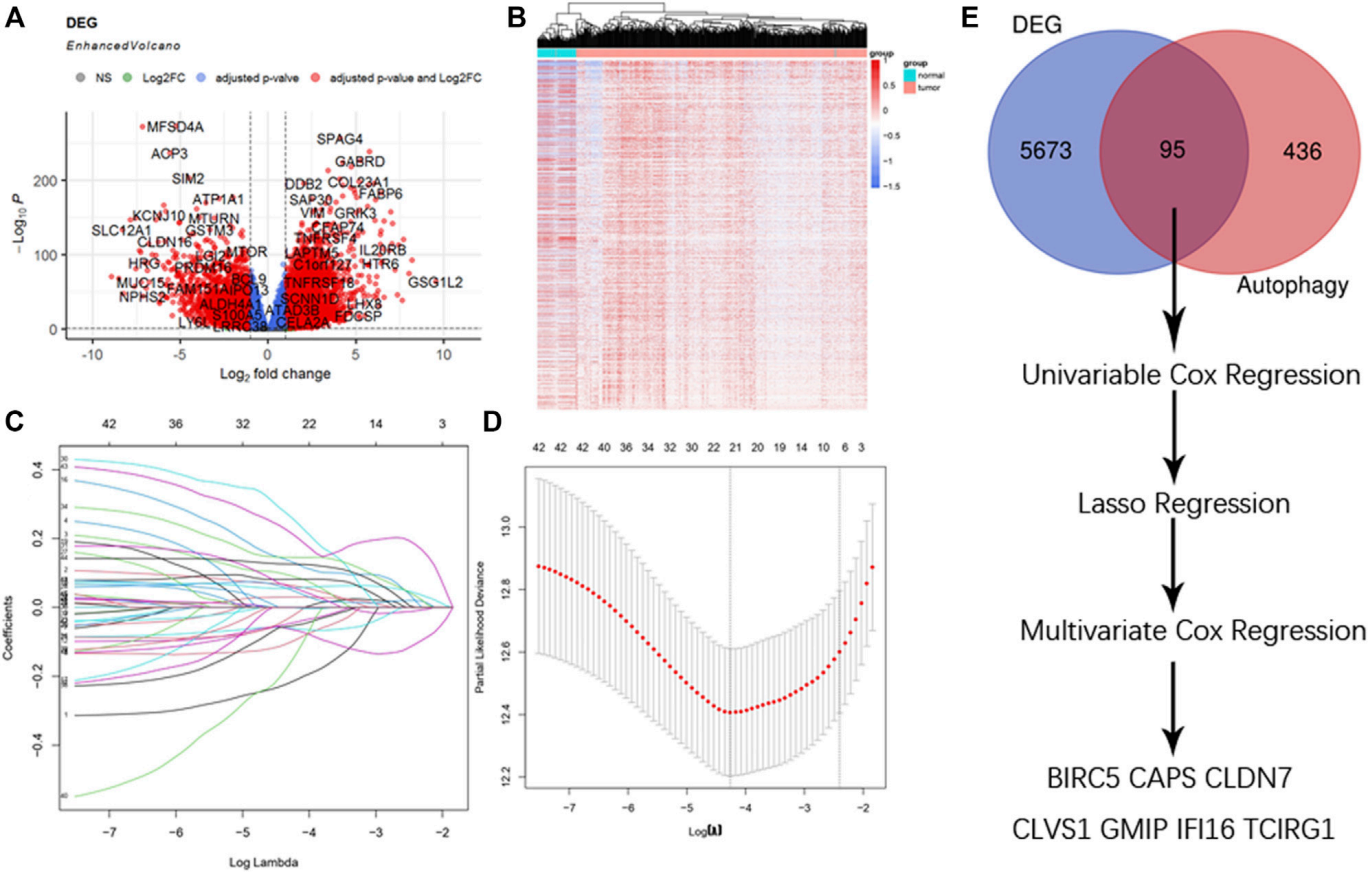
Selection of DEGs. **(A)** Enhanced volcano plot of DEGs when comparing ccRCC with normal tissue. Red nodes represented genes with |log_2_FC| ≥ 1 and adjusted *p* < 0.05, blue nodes represented genes with adjusted *p* < 0.05 only, and grey nodes represented genes that were neither eligible in conditions of adjusted *p* value nor |log_2_FC|. **(B)** Heatmap of DEGs in ccRCC. **(C)** Lasso coefficients profiles of 95 genes significant in univariate cox regression. **(D)** Lasso regression obtained 21 prognostic genes using minimum lambda value. **(E)** Selecting procedure of DEGs, venn gram showed 95 genes in the intersection of 5,768 DEGs and 531 ATGs. These genes then underwent univariate cox regression, lasso cox regression, multivariate cox regression, and finally 7 DEGs were selected to construct the risk score formula.

The raw counts data was downloaded through R package “TCGAbiolinks” from TCGA-KIRC with setting data category as “Transcriptome Profiling” and data type as “Gene expression Quantigication.” Up to 56,612 genes were downloaded, and after gene ID conversion we obtained 18,569 mRNA. They were then estimated with differential analysis between ccRCC patients and paracancerous patients by R package “Deseq2.” With the threshold of |logFC| ≥ 1, adjusted *p* value < 0.05, we obtained 5,768 DEGs among which 471 were up-regulated, 388 were down-regulated, and volcano plot and heatmap were drawn for better understanding (Figures 1A,B).

### Selection of DEGs

A total of 95 genes were in the intersection between DEGs and ATGs, and they were analyzed with univariate cox regression. Among 95 genes there were 46 genes with *p* < 0.05. We then performed lasso regression analysis and got 21 genes (Figures 1C,D). These 21 genes were analyzed by multivariate cox regression, among which 7 genes were found significant (*p* < 0.05). Thus, we obtained 7 genes (BIRC5, CAPS, CLDN7, CLVS1, GMIP, IFI16, and TCIRG1) as DEGs to construct the formula of new risk score (Figure 1E; Table1).

**TABLE 1:** Multivariate cox regression of DEGs.

Gene symbol	Coefficient	*p* Value	HR (95%CI)
BIRC5	0.000355	0.010	1.00036 (1.00009, 1.00062)
CAPS	0.000382	0.014	1.00038 (1.00008, 1.00069)
CLDN7	−0.000131	0.010	0.99987 (0.99977, 0.99997)
CLVS1	0.001747	<0.001	1.00175 (1.00105, 1.00245)
GMIP	−0.00033	0.048	0.99967 (0.99934, 1.00000)
IFI16	0.000082	0.007	1.00008 (1.00002, 1.00014)
TCIRG1	0.000118	0.021	1.00012 (1.00002, 1.00022)

HR, hazard ratio.

### Analysis of mRNA and Protein Expression Levels of DEGs

**FIGURE 2:**
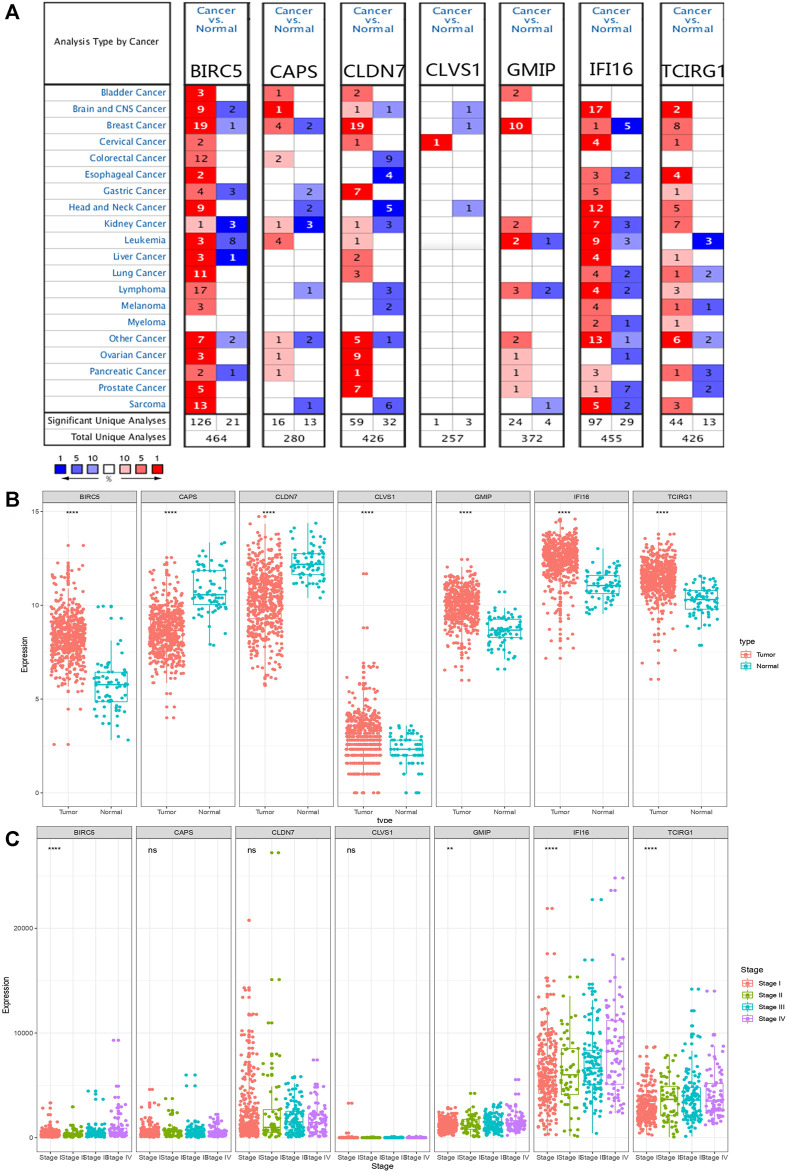
mRNA expression levels of DEGs. **(A)** mRNA expression levels of DEGs from ONCOMINE database. The threshold (*p* < 0.05, |log_2_FC| ≥ 1.5, gene rank: Top 10% datatype: mRNA) was indicated in the colored cells. The red cells indicated the target gene was overexpressed in ccRCC while blue cells represented downregulated in ccRCC. Gene rank was depicted in the color depth in the cells. **(B)** mRNA expression levels of DEGs in ccRCC and normal tissue from TCGA database. *p* value was replaced by “*,” ^ns^ *p* > 0.05, *p < 0.05, **p < 0.01, ***p < 0.001 and ****p < 0.0001. **(C)** Different mRNA expression levels of DEGs in different stages.

Comparing the mRNA expression levels of BIRC5, CAPS, CLDN7, CLVS1, GMIP, IFI16, and TCIRG1 in ccRCC patients and normal people, we found that BIRC5, CLVS1, GMIP, IFI16, and TCIRG1 were significantly overexpressed in ccRCC patients from TCGA-KIRC dataset and CAPS and CLDN7 had lower expression level in ccRCC patients than in normal people. Apart from BIRC5, GMIP, IFI16, and TCIRG1, other genes didn’t show significant differences in individual cancer stages (Figures 2A–C). Results from ONCOMINE database partly coordinated with what we found before, that BIRC5 was found overexpressed in Gumz Renal (*FC* = 2.753, *p* value < 0.001), IFI16 was highly expressed in ccRCC patients from Gumz Renal (*FC* = 4.454, *p* < 0.001), Yusenko Renal (*FC* = 5.099, *p* < 0.001), Lenburg Renal (*FC* = 2.160, *p* < 0.001) and Jones Renal (*FC* = 3.863, *p* < 0.001). GMIP was overexpressed in Yusenko Renal (*FC* = 4.020, *p* < 0.001). TCIRG1 was overexpressed in Yusenko Renal (*FC* = 2.516, *p* = 0.001), Jones Renal (*FC* = 2.153. *p* < 0.001), and Lenburg Renal (*FC* = 1.860, *p* = 0.001) (Supplementary Material S1).

**FIGURE 3:**
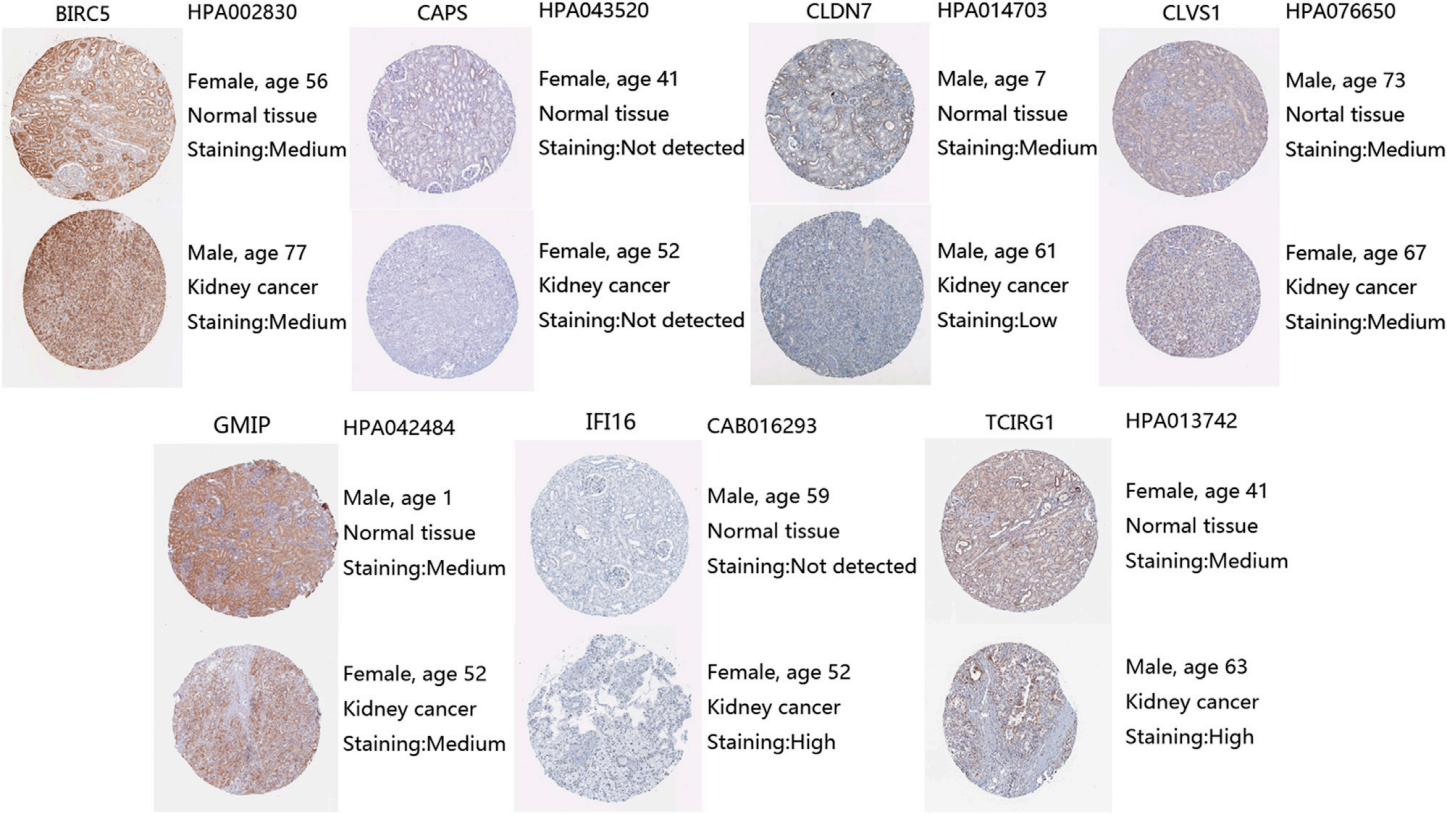
Protein expression levels of DEGs from HPA, antibody, and patient information were listed as well.

Moreover, we explored protein expression levels of DEGs in the HPA website, compared with normal kidney tissue, IFI16 and TCIRG1 were highly expressed in ccRCC kidney tissue. CLDN7 were found lower expressed in ccRCC tissue than in normal tissue. BIRC5, CLVS1, and GMIP were found the same level in ccRCC tissue as in normal tissue. Unfortunately, CAPS was not detected in ccRCC kidney tissue nor normal kidney tissue (Figure 3).

### PPI Network and Functional Enrichment Analysis

**FIGURE 4:**
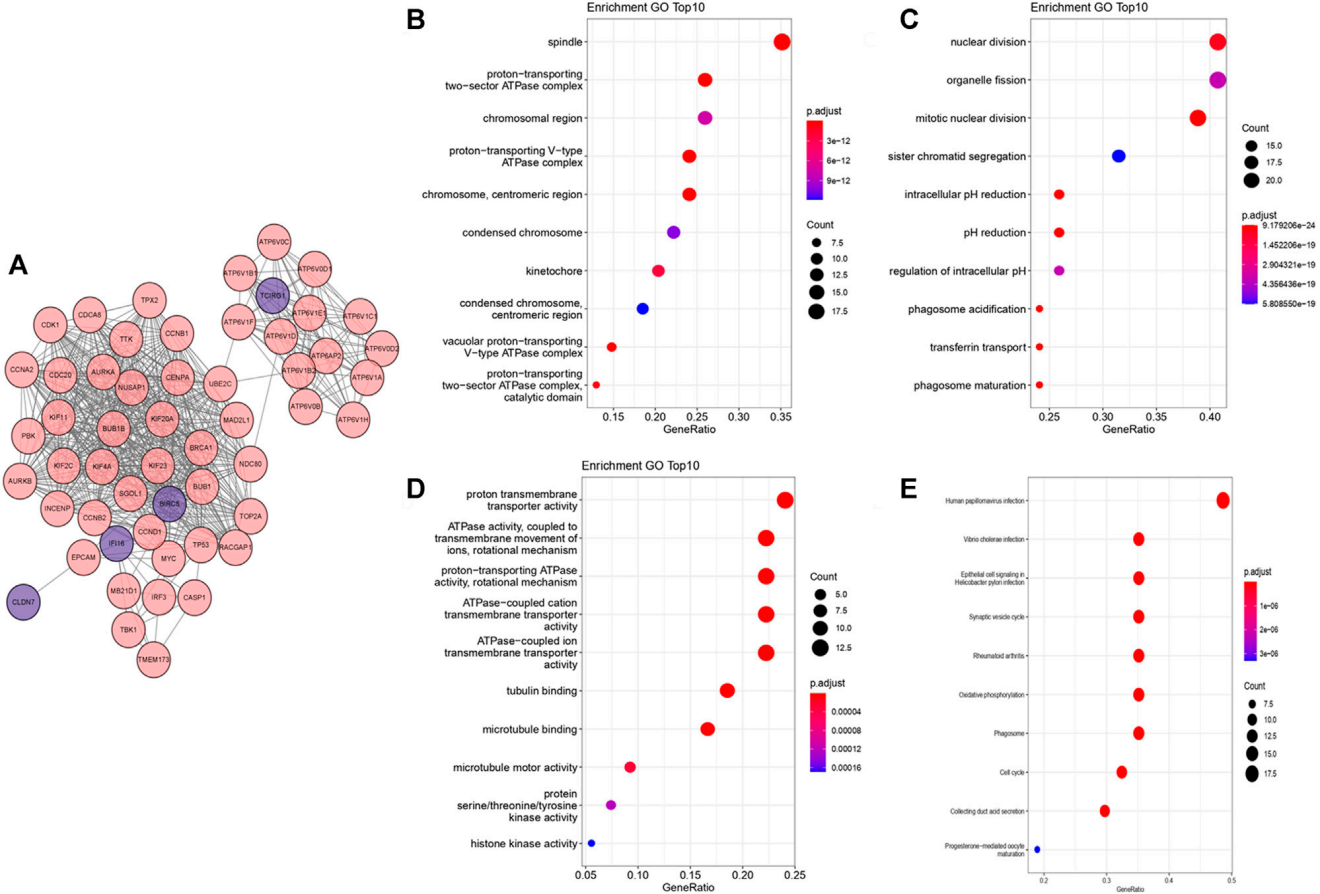
PPI network and functional enrichment analysis from “Clusterprofiler.” The deeper the color is, the more significant the enrichment is. The bigger the bubble is, the more genes are participated in the term. **(A)** PPI network from STRING visualized by cytoscape. **(B)** Bubble map of top 10 enriched GO terms in CC. **(C)** Bubble map of top 10 enriched GO terms in BP. **(D)** Bubble map of top 10 enriched GO terms in MF. **(E)** Bubble map of top 10 KEGG enriched pathways.

We performed PPI network in STRING and selected the top 50 closest genes with DEGs (Supplementary Material S2, Figure 4A). After GO function enrichment analysis and KEGG pathway analysis, we found the top 10 significant items of 57 genes in cellular component (CC) were spindle, protein-transporting two-secor ATPase complex, chromosomal region, proton-transporting V-type ATPase complex, chromosome, centromeric region, condensed chromosome, kinetochore, condensed chromosome, centromeric region, vacuolar protein-transporting V-type ATPase complex, proton-transporting two-sector ATPase complex, and catalytic domain (Figure 4B). The top 10 significant items of biological process (BP) included nuclear division, organelle fission, mitotic nuclear division, sister chromatid segregation, intracellular pH reduction, pH reduction, regulation of intracellular pH, phagosome acidification, transferrin transport, and phagosome maturation (Figure 4C). Top molecular functions (MFs) were mainly associated with energy metabolism including proton transmembrane transporter activity, ATPase activity, coupled to transmembrane movement of ions, rotational mechanism, ATPase-coupled cation transmembrane transporter activity, ATPase-coupled ion transmembrane transporter activity, tubulin binding, microtubule binding, microtubule motor binding, protein serine/threonine/tyrosine kinase activity, and histone kinase activity (Figure 4D). In KEGG pathway analysis, several pathways were found related with autophagy, such as rheumatoid arthritis, phagosome, cell cycle, and oxidative phosphorylation (Figure 4E).

### Construction of Risk Score

According to expression levels of 7 DEGs and their coefficients, we constructed the formula of risk score (Table 1):

$$\mathrm{Risk}\;\mathrm{score}=\sum_{i=1}^{7}coef_{i}*Exp_{i}$$

**FIGURE 5:**
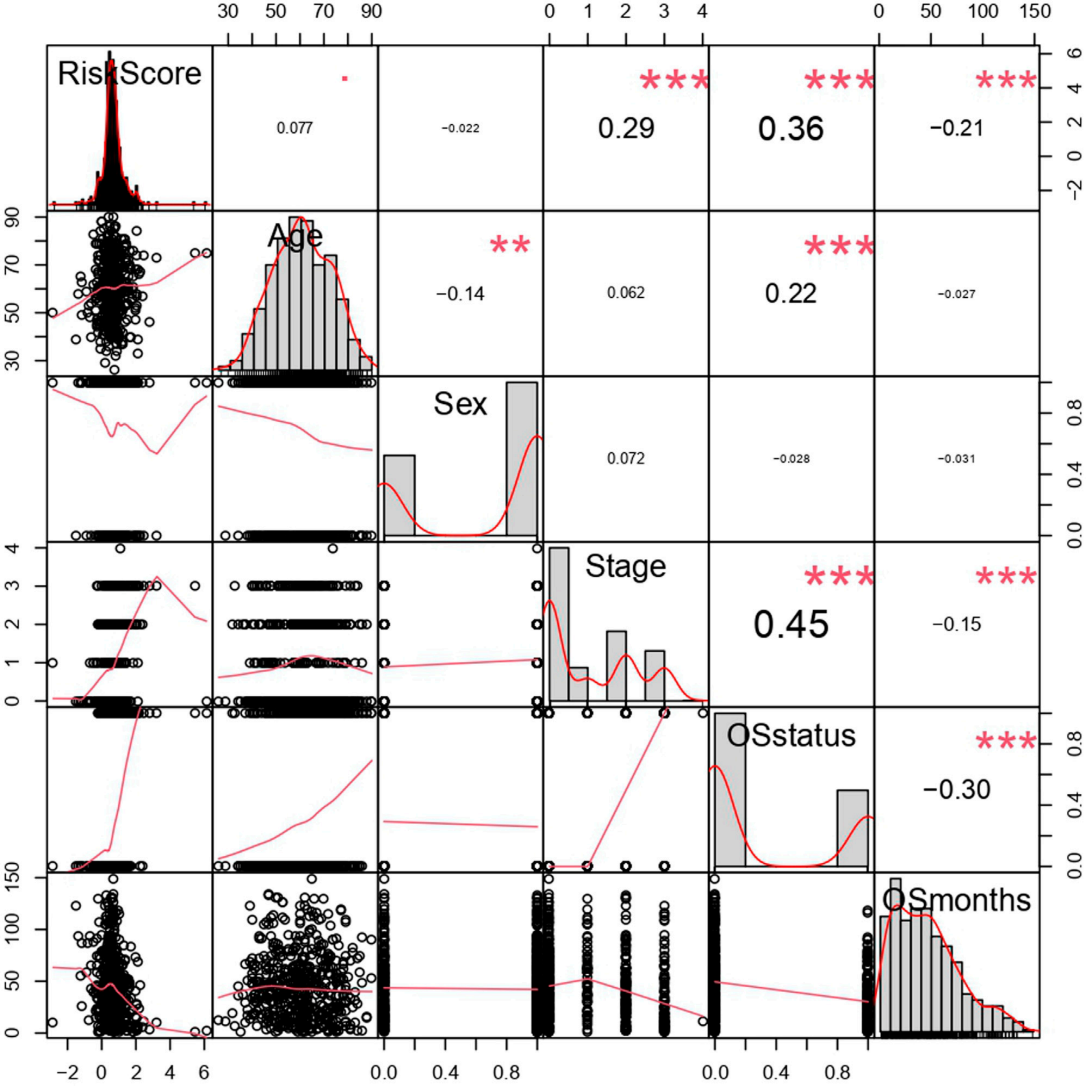
Correlation analysis of risk score and clinical signatures. **p < 0.01, ***p < 0.001, coefficients> 0 represented positive correlation while coefficients <0 represented negative correlation. The bigger the |coefficient| is, the more relevant the terms were.

**FIGURE 6:**
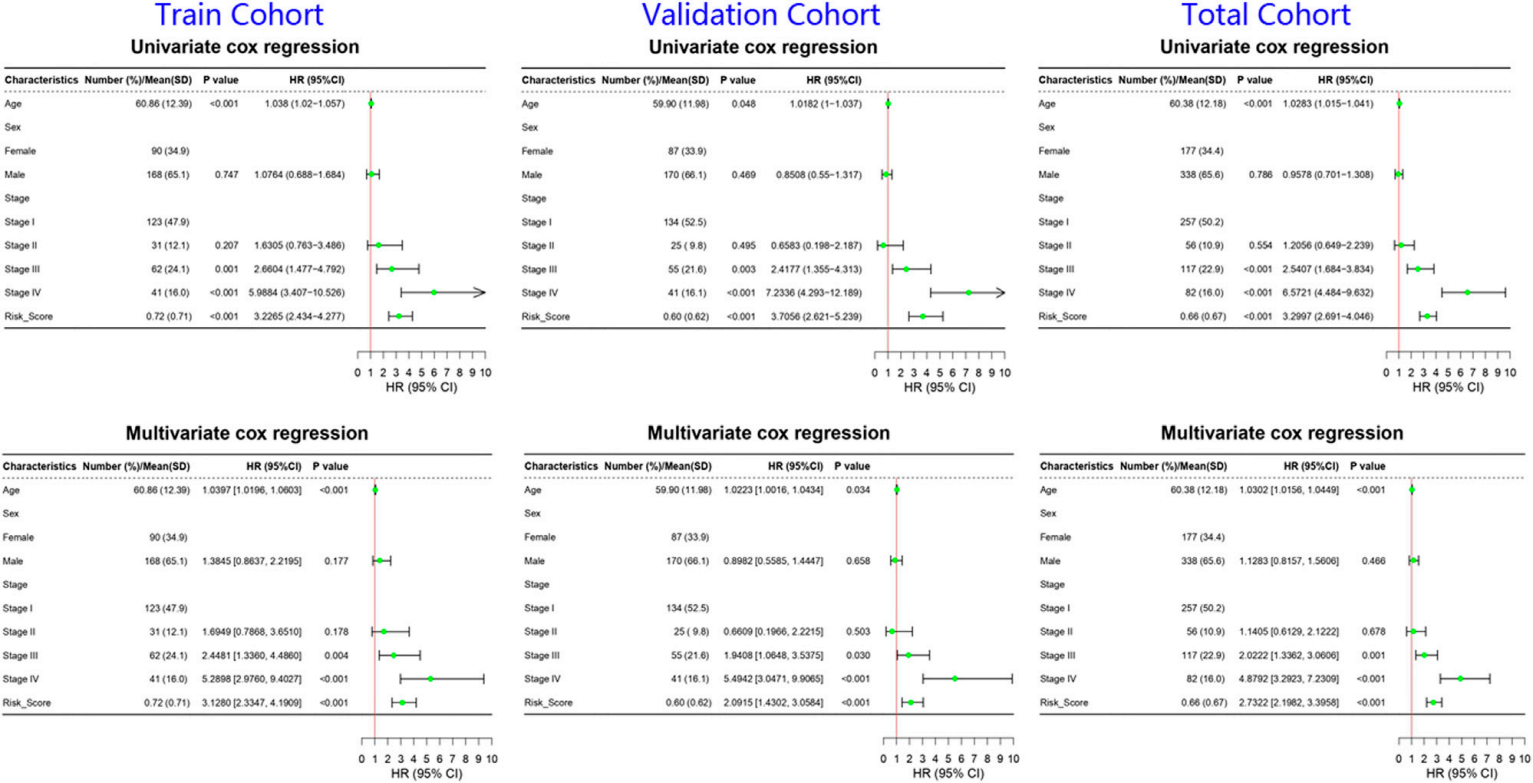
Forrest plots of univariate and multivariate cox regression of ccRCC patients in train cohort, validation cohort, and total cohort. The green nodes represented HR and the line extending from the nodes indicated 95% CI.

**FIGURE 7:**
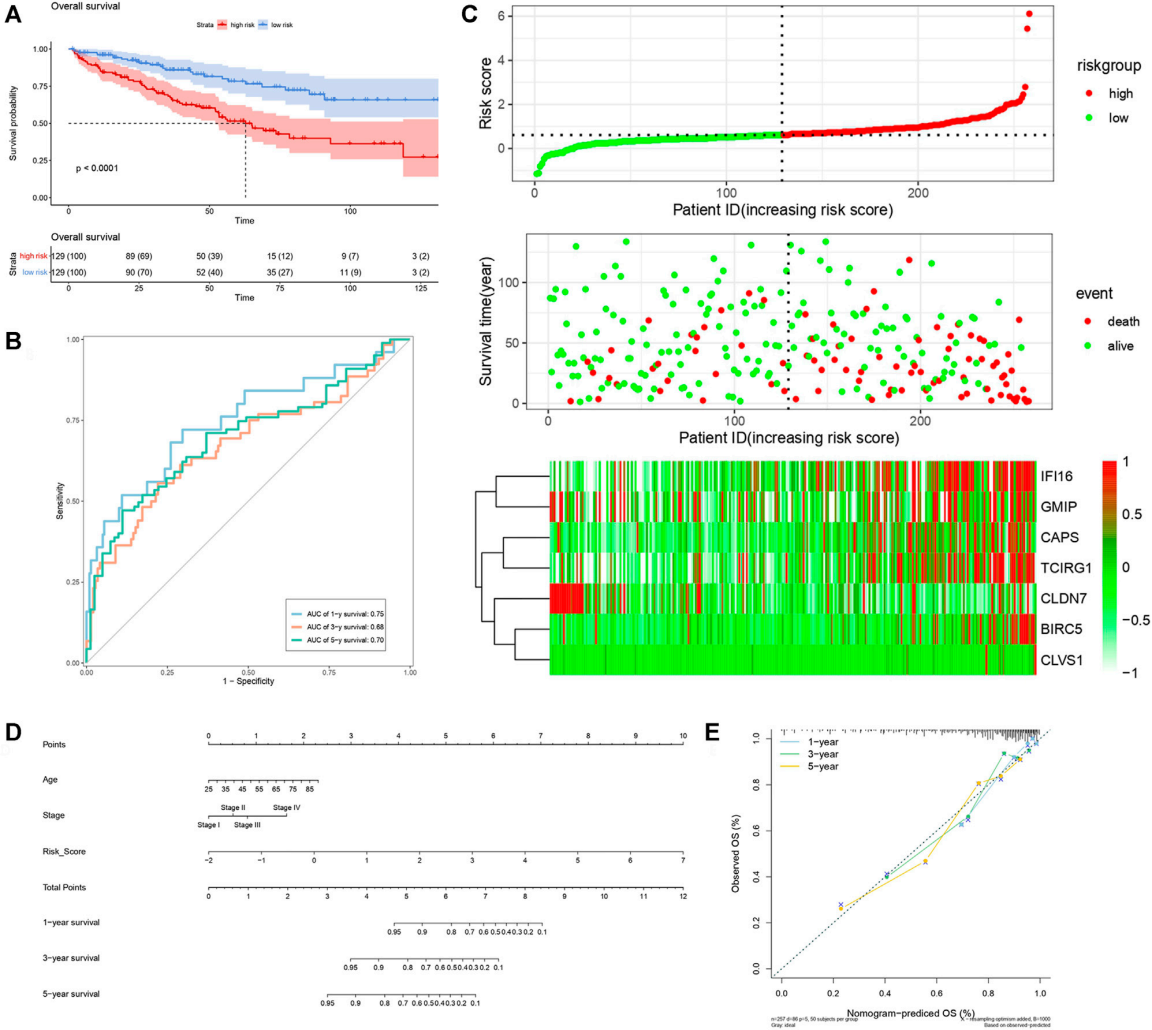
Verification of risk score as predicting factor of ccRCC patients in train cohort. **(A)** Survival curve of different groups. Red line represented patients in high risk group, blue line represented patients in low risk group. **(B)** Time-dependent ROC curve of 1-, 3-, 5-years. **(C)** Risk factor association diagram. Red nodes in the upper and middle graph represented patients with high risk score, green nodes represented patients with low risk score. Cells in the gram below represented each patients and color of cells indicated up- or down-regulation of genes. **(D)** Nomogram included age, stage, and 7 gene-based risk score. **(E)** Calibration curve was depicted for verification accurancy of nomogram predicting 1-, 3-, 5-years OS rate.

**FIGURE 8:**
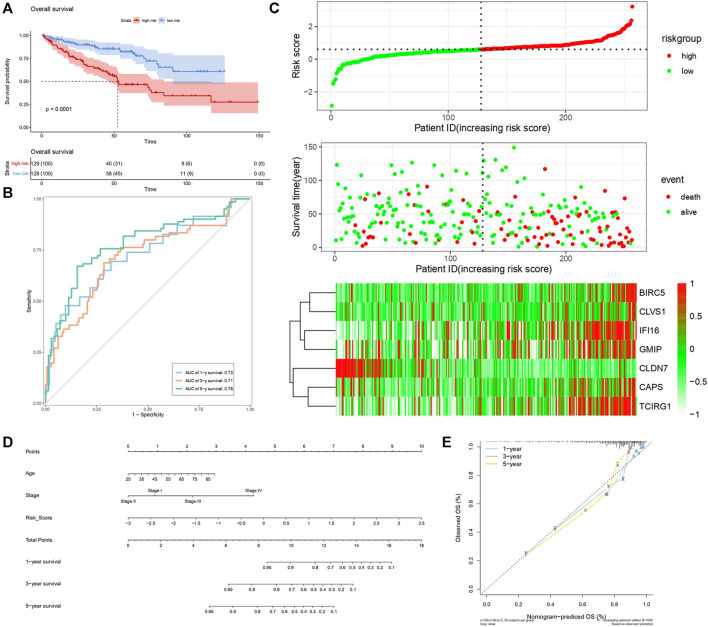
Verification of risk score as predicting factor of ccRCC patients in validation cohort. **(A)** Survival curve of different groups. Red line represented patients in high risk group, blue line represented patients in low risk group. **(B)** Time-dependent ROC curve of 1-, 3-, 5-years. **(C)** Risk factor association diagram. Red nodes in the upper and middle graph represented patients with high risk score, green nodes represented patients with low risk score. Cells in the gram below represented each patient, and color of cells indicated up- or down-regulation of genes. **(D)** Nomogram included age, stage, and 7 gene-based risk score. **(E)** Calibration curve was depicted for verification accurancy of nomogram predicting 1-, 3-, 5-years OS rate.

**FIGURE 9:**
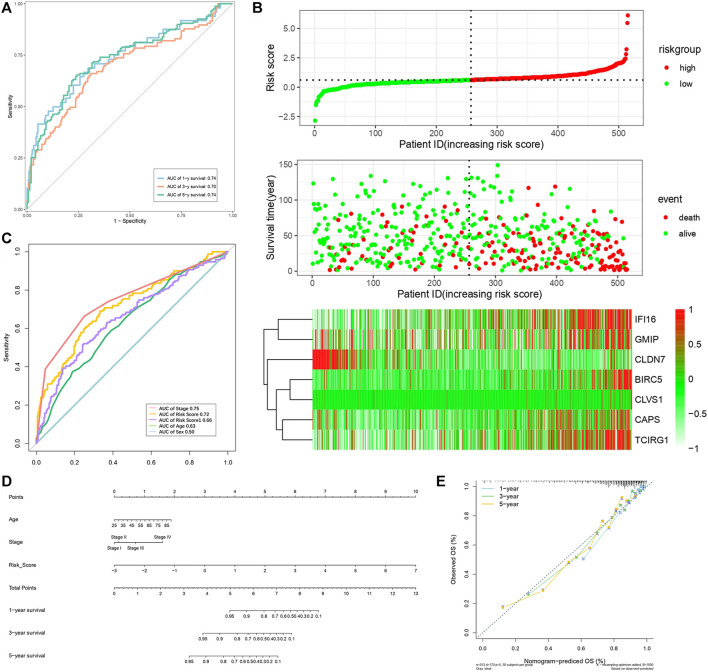
Verification of risk score as predicting factor of ccRCC patients in total cohort. **(A)** Time-dependent ROC curve of 1-, 3-, 5-year. **(B)** ROC curve of clinical signatures of ccRCC patints. **(C)** Risk factor association diagram. Red nodes in the upper and middle graph represented patients with high risk score, green nodes represented patients with low risk score. Cells in the gram below represented each patients and color of cells indicated up- or down-regulation of genes. **(D)** Nomogram included age, stage, and 7 gene-based risk score. **(E)** Calibration curve was depicted for verification accurancy of nomogram predicting 1-, 3-,5-years OS rate.

**FIGURE 10:**
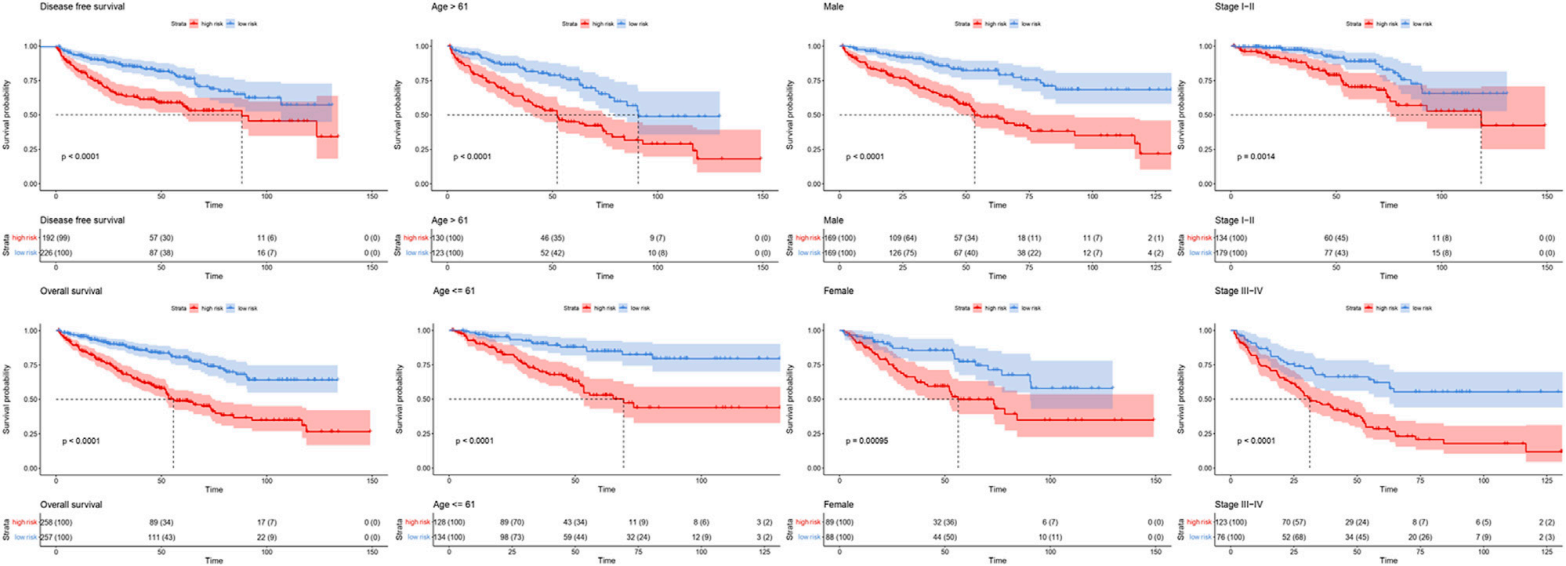
Survival curve of high and low risk groups in different cohorts sorted by clinical signatures. Red line represented patients in high risk group, blue line represented patients in low risk group.

By analyzing correlation between risk score and other clinical signatures, we found that risk score was positively associated with stage and OS status while negatively related with OS months (Figure 5). Analyzing clinical characteristics and risk score of patients from TCGA-KIRC in train cohort, validation cohort, and total cohort, we found age of diagnosis, stage, and risk score were independent risk factors of prognosis of ccRCC patients (Figure 6). Dividing all patients from train cohort into two groups (high risk and low risk) according to median of risk score, K-M curve, and Log-rank test of OS demonstrated significant difference, and the high risk group had shorter OS than patients in low risk group (Figure 7A). Time-dependent ROC curve showed the AUC of the first year, the third year, and the fifth year was 0.75, 0.68, and 0.70, respectively, which indicated risk score had a good predictive ability (Figure 7B). Depicting risk factor association diagram, it was clear to see that as risk score rose, mortality grew, and survival time was reduced (Figure 7C). Combining with all the clinical signatures that mattered, we constructed a nomogram to predict the survival rate of ccRCC patients. Calibration curve verified the accuracy of its ability to predict prognosis (Figures 7D,E). Similar analyses were performed in the validation cohort which provided stronger evidence of our risk score having significant value in predicting prognosis of ccRCC patients with AUC of the first year, the third year, and the fifth year of 0.73, 0.71, and 0.78, respectively. Nomogram and calibration in validation cohort also validated the practicability of the model (Figures 8A–E). In the total cohort, time-dependent ROC curve showed the ability of risk score predicting prognosis with AUC of the first year, the third year, and the fifth year at 0.74, 0.70, and 0.74, respectively (Figure 9A). Comparing with other clinical signatures we found the AUC of stage was 0.75, AUC of risk score was 0.72, AUC of age was 0.63, and AUC of sex was 0.50. We’ve published a glycolysis-related risk score before, and since they were both metabolic-related signatures we compared their ability of predicting prognosis of ccRCC patients by AUC. It turned out that as the AUC of glycolysis-related risk score was 0.66, the autophagy-related risk score had better ability of prediction (Figure 9B). Risk factor association diagram showed as risk score rose, mortality grew, and survival time reduced (Figure 9C). Calibration of nomogram of total cohort perfectly consisted with results in train cohort and validation cohort (Figures 9D,E). Moreover, K-M curves indicated patients in the low risk group had longer OS and DFS than in the high risk group. In addition, to eliminate influences from clinical characteristics, we grouped all the patients by age, gender, and stage and proved significant difference in survival time for patients with different levels of risk score (Figure 10). Thus, although not as efficient as stage, risk score could still be a reliable index to predict prognosis of ccRCC patients without concern about clinical characteristics.

**FIGURE 11:**
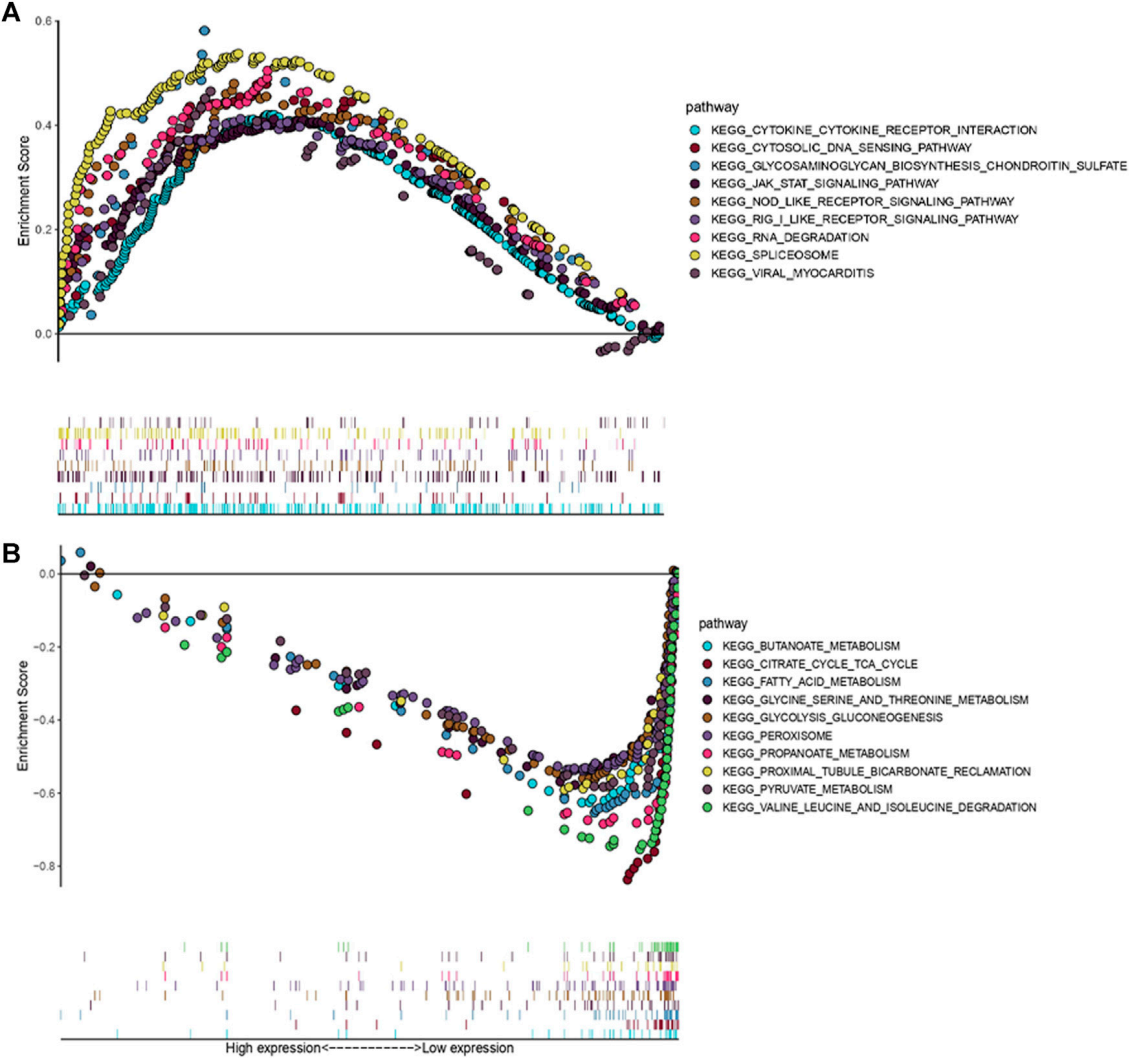
GSEA associated with risk score [Gene matrix: c2.cp.kehh.v7.symbols.gmt (Curated), Number of permutations: 1,000, Permutation type: phenotype]. **(A)** KEGG pathways enriched in high risk group. **(B)** KEGG pathways enriched in low risk group.

**FIGURE 12:**
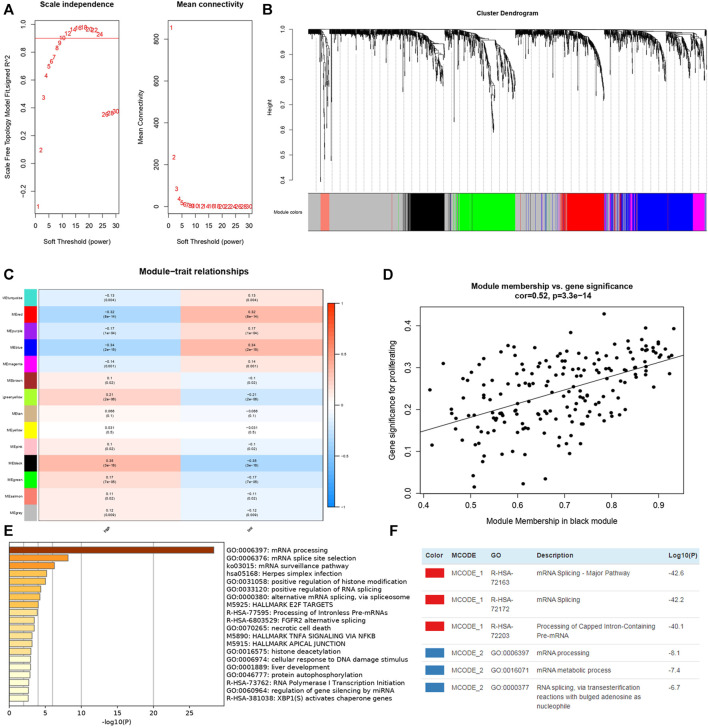
WGCNA in TCGA-KIRC dataset and enrichment analysis of genes in black module from Metascape. **(A)** Soft thresholding filtering. **(B)** Module screening, 14 modules have been identified. **(C)** The relationship between 14 module and different risk groups, the red cells indicated the module was positively related with high/low risk group while blue cells represented negative relationship. **(D)** Correlation plot between genes in black module and high risk score. **(E)** Top 20 pathways enriched in black module. **(F)** MCODEs of black module.

In order to figure out different signatures underlying two risk groups, we further performed GSEA and WGCNA analysis, respectively. Dividing patients into high and low risk groups, a total of 18,569 genes were analyzed through GSEA. We regarded KEGG pathways with |NES| ≥ 1.5, FDR < 0.25 as significant, and the results indicated the high risk group was connected with cytokine-cytokine receptor interaction, cytosolic DNA sensing pathway, glycosaminoglycan biosynthesis chondroitin sulfate, JAK-STAT signaling pathway, NOD-like receptor signaling pathway, RIG-I like receptor signaling pathway, RNA degradation, spliceosome, and viral myocarditis (Figure 11A). Meanwhile, butanoate metabolism, citrate cycle tca cycle, fatty acid metabolism, glycine serine and threonine metabolism, glycolysis gluconeogenesis, peroxisome, propanoate metabolism, proximal tubule bicarbonate reclamation, pyruvate metabolism and valine leucine, and isoleucine degradation were found enriched in the low risk group (Figure 11B). Genes with top 5,000 median absolute deviation were analyzed in WGCNA, and soft threshold was set as 9 (Figure 12A). Correlation between risk score and modules was calculated, as the figure shows that a black module was related with high risk score closely (Figures 12B,C). As a result, we analyzed the relation among genes in black module and high risk score and found they were positively related (cor = 0.52, *p* < 0.001) (Figure 12D). Finally, we performed functional enrichment analysis in Metascape, with the threshold of min overlap = 3, *p* value cutoff = 0.01, and min enrichment = 1.5, and 20 pathways were found enriched in black module (Figure 12E). MCODE was selected with physical score > 0.132, min network size = 3, max network size = 500, and databases as physical core (Figure 12F).

### Immune Cell Infiltration

**FIGURE 13:**
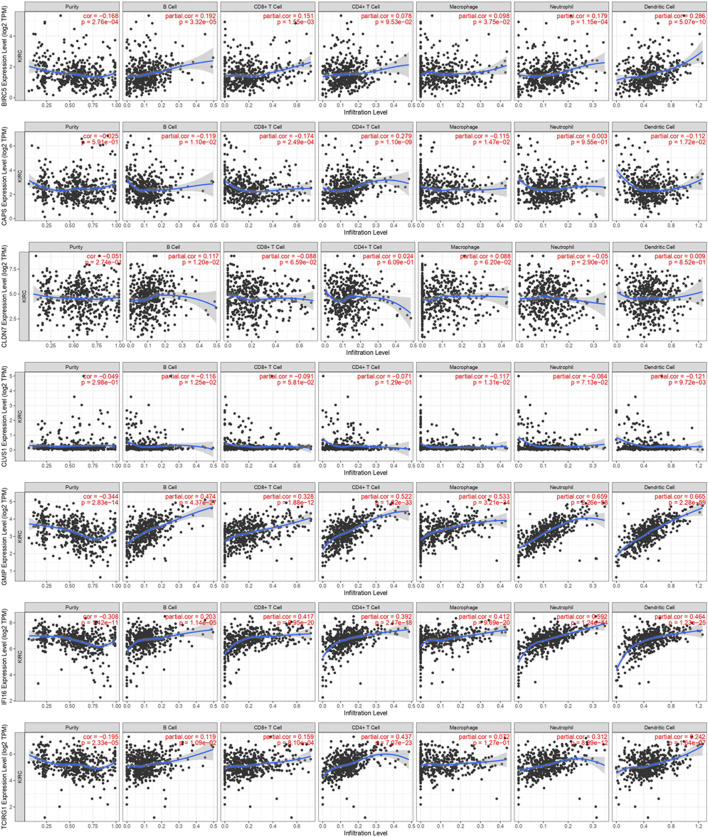
Immune cell infiltration of 7 DEGs in TCGA-KIRC from TIMER.

Concerning the significance of immunity on tumorigenesis and progression, we analyzed immune cell infiltration of these seven DEGs on TIMER (Figure 13). We found expression of BIRC5 was positively associated with B Cell, CD8^+^ T Cell, Marcophage, Neutrophill and Dendritic Cell. CAPS was negatively associated with B Cell, CD8^+^ T Cell, Marcophage and Dendritic Cell and positively associated with CD4^+^ T Cell. CLDN7 was found positively related with B Cell, and CLVS1 was found negatively related with B Cell, Macrophage and Dendritic Cell. High expression levels of GMIP and IFI16 were highly related with B Cell, CD8^+^ T Cell, CD4^+^ T Cell, Marcophage, Neutrophill and Dendritic Cell. Similarly, TCIRG1 was found positively related with B Cell, CD8^+^ T Cell, CD4^+^ T Cell, Neutrophill and Dendritic Cell.

**FIGURE 14:**
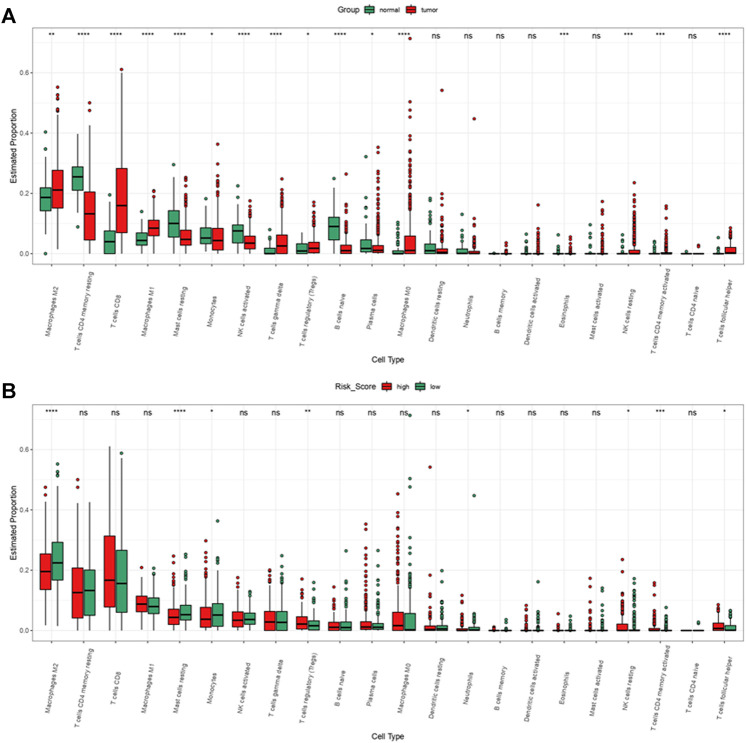
Immune cell infiltration of ccRCC patients from TCGA-KIRC dataset. **(A)** Immune cell infiltration levels in ccRCC and normal tissue. **(B)** Immune cell infiltration levels of high and low risk group.

We performed “Cibersort” on R studio to assess immune cell infiltration level in ccRCC patients and normal patients, macrophages M2, T cells CD8, macrophages M1, T cells gamma delta, T Cells regulatory, macrophages M0, NK cells resting, T cells CD4 activated and T cells follicular are significantly higher infiltrated in ccRCC patients than in normal patients (Figure 14A). Meanwhile, comparing immune cell infiltration level between high risk group and low risk group, we found macrophages M2, monocytes, mast cells resting, and neutrophils were significantly lower infiltrated in high risk group, NK cell resting, T cells CD4 memory activated, and T cells regulatory and T cells follicular helper were infiltrated significantly high in high risk group (Figure 14B). It seemed that immune cells highly enriched in high risk group and ccRCC patients were not typical immune cells that promoted tumorigenesis and progression. As a result, to find out immune cells that had significant effects on ccRCC, we performed univariate and multivariate cox regression on immune cell infiltration and the results revealed that in TCGA-KIRC, mast cells resting suppressed tumor progression while macrophages M0, T cells CD4 memory activated, and T cells regulation were risk factors of tumor progression (Supplementary Material S3).

## Discussion

Autophagy is an indispensable biological process which enables cells to self-degrade and recycle intracellular components. It is well-recognized that in the early stage of tumorigenesis, autophagy represses tumorigenesis by its function of stability and inhibiting genome destruction from metabolic stress and immunoreaction (Karantza-Wadsworth et al., 2007; White, 2012). On the other hand, in the late stage autophagy protects tumor cells from stress to improve tumor progression. Studies indicated basic function of autophagy provides cellular metabolites for tumor cells and regulates mitochondrial metabolism to meet the high metabolic requirements of rapid proliferation of tumor cells (White, 2012; Katheder et al., 2017). In addition, autophagy not only modulates transfer-related biological phenotypes such as resistance to anoikis (Coates et al., 2010), but also stimulates TGF-β and EMT process (Li et al., 2013; Papageorgis, 2015; Yeo et al., 2016). Thus it can be seen that autophagy affects on tumorigenesis and tumor progression through multiple approaches. In our study we aim to explore how autophagy affects progression of ccRCC and seek ATGs that can predict the progression of ccRCC.

Through lasso regression analysis and cox regression analysis, we finally identified 7ATGs (BIRC5, CAPS, CLDN7, CLVS1, GMIP, IFI16, and TCIRG1) associated closely with ccRCC prognosis. BIRC5 is a EMT related gene which prevents cell apoptotic through different approaches and participates in cell cycle regulation, and also in cancer cells it regulates autophagy directly (Lin et al., 2020). High expression of BIRC5 was found to indicate poor prognosis in hepatocellular carcinoma (Xu et al., 2021). Also BIRC5 was found related with prognosis of ccRCC and gastric cancer (Yao et al., 2020; Li et al., 2021). CAPS encodes a calcium-binding protein, which may play a role in the regulation of ion transport; research showed that CAPS might indicate tamoxifen resistance in ER positive breast cancer (Johansson et al., 2015). CLDN7 encodes a member of claudin family and were found expressed in several malignancies such as prostate cancer, lung cancer, urinary tumors, and so on. Overexpression of CLDN7 is closely related to lymph node metastasis (Wu et al., 2018). In addition, CLDN7 was found upregulated in mouse pancreas exposed to caerylein for 12 h and its function concerned tight junction formation, while destruction of tight might be closely related with autophagy’s detrimental effects (Nakada et al., 2010; Wang S. et al., 2020). So far CLVS1 wasn’t found significant in tumorigenesis and progression, but research found it is involved in lysosome maturation and associated with psychiatric and steroid-sensitive nephrotic syndrome (Corponi et al., 2019; Lane et al., 2021). GMIP is a protein coding gene that encodes ARHGAP family of Rho/Rac/Cdc42-like GTPase activating proteins. In lung cancer, overexpression of GMIP was associated with longer survival; in the null mice model with a xenografted tumor of A549 cells, GMIP treatment has once been proved to induce autophagy and reduce tumor growth (Hsin et al., 2011; Amaar and Reeves, 2020). IFI16 modulates p53 function and inhibits cell growth in the Ras/Raf signaling pathway. It can be induced by AMPK/p53 pathway and the induced levels of IFI16 were associated with the induction of autophagy (Duan et al., 2011). TCIRG1 is involved in autophagosome assembly, and it is usually found relevant with osteopetrosis (Belaid et al., 2013; Chavez-Guitron et al., 2018).

We first analyzed expression levels in ccRCC of these seven genes, and in mRNA level, results from ONCOMINE showed except GMIP, IFI16, and TCIRG1 were overexpressed in ccRCC patients, while analysis based on TCGA indicated expression levels of CAPS and CLDN7 were significantly low in ccRCC patients, and BIRC5, CLVS1, GMIP, IFI16, and TCIRG1 were highly expressed in ccRCC patients. In protein level, we found, IFI16, and TCIRG1 were highly expressed in ccRCC kidney tissue, and others were lower in ccRCC kidney tissue or not detected. Through STRING we found the top 50 related genes of these 7 genes and performed functional enrichment and pathway analysis, and results revealed they were closely related with autophagy process, tumorigenesis, and involved in biological processes of tumor progression. Results from GSEA analyzing functional enrichment of high and low risk group indicated that JAK-STAT signaling pathway, NOD-like receptor signaling pathway, and RIG-I-like receptor signaling pathway might be the cause of poorer prognosis of ccRCC patients (Smith et al., 2018; Mey et al., 2019; Zhou et al., 2020). Actually, these pathways were found directly or indirectly related with autophagy (Chan and Gack, 2015; Velloso et al., 2019; Billah et al., 2020). Further, we explored a module co-expressed with high risk in WGCNA, and genes from the most significant module were found quite closely connected with high risk, too. Functional enrichment analysis was performed in Metascape then.

According to the formula we construct a new ATGs-related risk score in train cohort, and we found the high risk score was related to poor prognosis of ccRCC patients. Cox regression analysis indicated together with age of diagnosis and stage, risk score was an independent risk factor of prognosis of ccRCC. All the results above were verified by similar analysis in validation cohort and total cohort. Concerned about the importance of immune response in tumorigenesis and progression, we further explored immune infiltration of 7 genes, and all of them were related with immune cells in varying degrees. The high risk group was highly infiltrated with NK cell resting, T cells CD4 memory activated, T cells regulatory, and T cells follicular helper.

Although all results above demonstrated that the risk score signatures constructed by 7 DEGs contribute to the progression of ccRCC patients and functional enrichment related with risk score demonstrated risk score had an association with autophagy, whether autophagy itself in our study took the responsibility of tumor progression remained unknown. Further experiments *in vivo* and *in vitro* are still needed to prove practicality and feasibility of the new risk score.

## Conclusion

A serious of analysis based on autophagy and DEGs was performed, and it turned out that a new risk score constructed by 7 ATGs (BIRC5, CAPS, CLDN7, CLVS1, GMIP, IFI16, and TCIRG1) could be a potential predictive signature of ccRCC patients. The relevant findings in this study still need mechanism and molecular verification in the future.

## Data Availability

Publicly available datasets were analyzed in this study. This data can be found here: https://portal.gdc.cancer.gov/ and remaining datasets are available in Supplementary Materials.
