# Corrigendum: A New Prognostic Risk Score: Based on the Analysis of Autophagy-Related Genes and Renal Cell Carcinoma

**DOI:** 10.3389/fgene.2022.904512

**Published:** 2022-07-04

**Authors:** Minxin He, Mingrui Li, Yibing Guan, Ziyan Wan, Juanhua Tian, Fangshi Xu, Haibin Zhou, Mei Gao, Hang Bi, Tie Chong

**Affiliations:** ^1^ Department of Urology, The Second Affiliated Hospital, School of Medicine, Xi’an Jiaotong University, Xi’an, China; ^2^ School of Medicine, Xi’an Jiaotong University, Xi’an, China

**Keywords:** risk score, prognosis, bioinformatics analysis, renal cell carcinoma, autophagy

In the original article, there was a mistake in the legend for Figure 1A. The threshold of DEGs was wrongly depicted in the legend. The correct legend is presented as follows:

**FIGURE 1:**
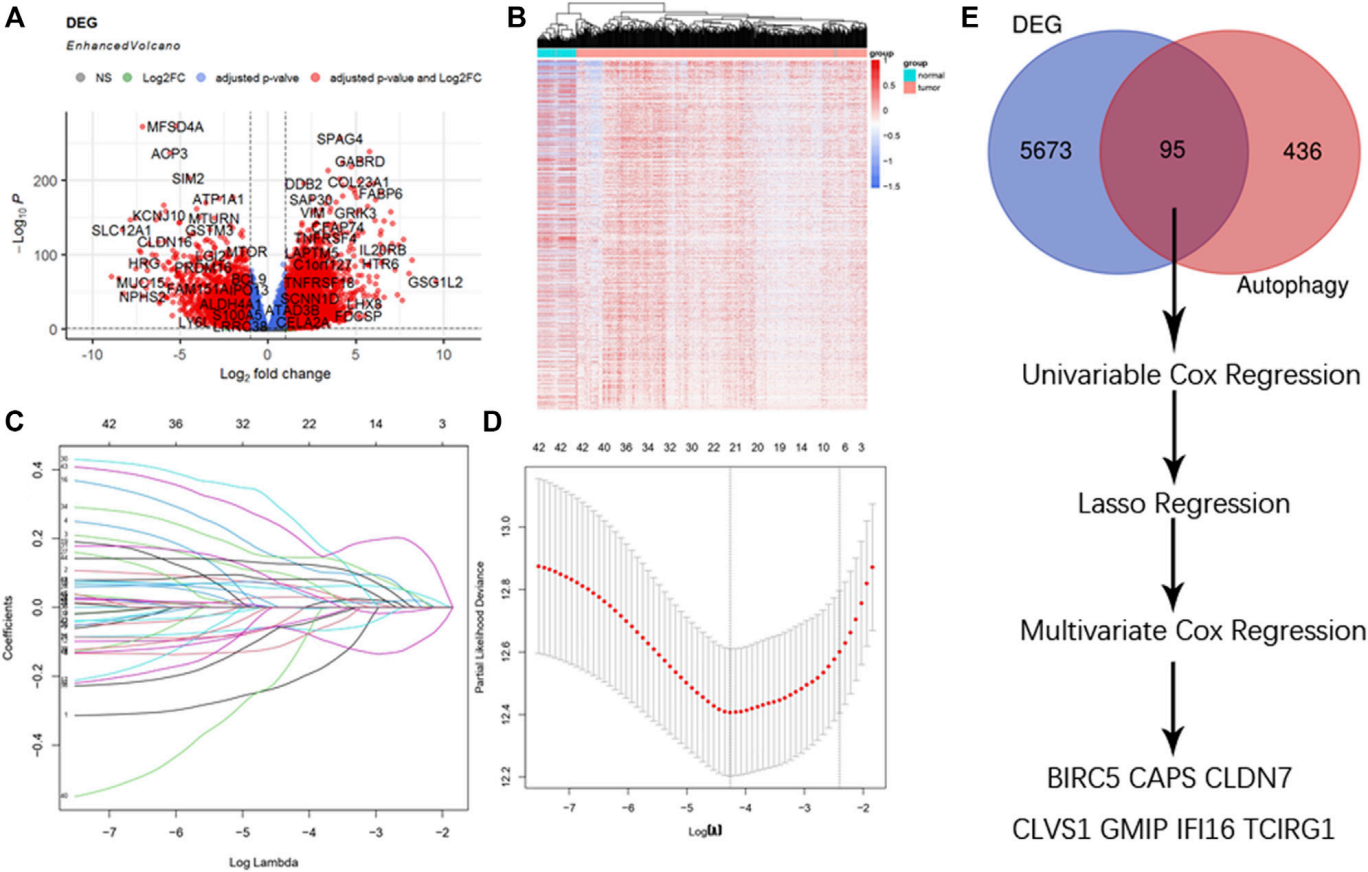
Selection of DEGs. **(A)** Enhanced volcano plot of DEGs when comparing ccRCC with normal tissue. Red nodes represented genes with |log 2 FC| ≥ 1 and adjusted *p* < 0.05, blue nodes represented genes with adjusted *p* < 0.05 only, and grey nodes represented genes that were neither eligible in conditions of adjusted *p* value nor |log 2 FC|. **(B)** Heatmap of DEGs in ccRCC. **(C)** Lasso coefficients profiles of 95 genes significant in univariate cox regression. **(D)** Lasso regression obtained 21 prognostic genes using minimum lambda value. **(E)** Selecting procedure of DEGs, venn gram showed 95 genes in the intersection of 5,768 DEGs and 531 ATGs. These genes then underwent univariate cox regression, lasso cox regression, and multivariate cox regression, and finally 7 DEGs were selected to construct the risk score formula.

“(A) Enhanced volcano plot of DEGs when comparing ccRCC with normal tissue. Red nodes represented genes with |log 2 FC| ≥ 1 and adjusted *p* < 0.05, blue nodes represented genes with adjusted *p* < 0.05 only, and gray nodes represented genes that were neither eligible in conditions of adjusted *p*-value nor |log 2 FC|.”

In the original article, there was a mistake in Figure 1 as published. The threshold of DEGs was wrongly set when drawing enhanced volcano plots the corrected Figure 1 is included here.

In the original article, the method of correlation analysis was wrongly typed as “pearson” in “Correlation analysis of risk score and other clinical signatures was performed by the method of “pearson”.” A correction has been made to **Materials and Methods, Construction of Risk Score**, Paragraph 2:

The sentence “Correlation analysis of risk score and other clinical signatures was performed by the method of “pearson”.” should be corrected as “Correlation analysis of risk score and other clinical signatures was performed by the method of “Spearman”.”

The authors apologize for this error and state that this does not change the scientific conclusions of the article in any way. The original article has been updated.

