# Supplementary material for: A New Prognostic Risk Score: Based on the Analysis of Autophagy-Related Genes and Renal Cell Carcinoma
Source: Front Genet. 2022 Feb 14;12:820154. doi: 10.3389/fgene.2021.820154 (PMC8884161; doi:10.3389/fgene.2021.820154)
Supplement: Supplementary file 3 [file Table1.DOCX]

mRNA expression levels of DEGs in different dataset from ONCOMINE

| Gene Symbol | Type | FC | P value | Dataset |
| --- | --- | --- | --- | --- |
| BIRC5 | Clear Cell Renal Cell Carcinoma | 2.753 | <0.001 | Gumz Renal |
| IFI16  GMIP  TCIRG1 | Clear Cell Renal Cell Carcinoma  Clear Cell Renal Cell Carcinoma  Clear Cell Renal Cell Carcinoma  Clear Cell Renal Cell Carcinoma  Clear Cell Renal Cell Carcinoma  Clear Cell Renal Cell Carcinoma  Clear Cell Renal Cell Carcinoma  Clear Cell Renal Cell Carcinoma | 4.454  5.099  2.160  3.863  4.020  2.153  1.860  2.516 | <0.001  <0.001  <0.001  <0.001  <0.001  <0.001  0.001  0.001 | Gumz Renal  Yusenko Renal  Lenburg Renal  Jones Renal  Yusenko Renal  Jones Renal  Lenburg Renal  Yusenko Renal |
